# Time‐Lapse Acquisition of Both Freely Secreted Proteome and Exosome Encapsulated Proteome in Live Organoids’ Microenvironment

**DOI:** 10.1002/advs.202406509

**Published:** 2024-11-21

**Authors:** Haoni Yan, Aynur Abdulla, Aiting Wang, Shuyu Ding, Manlin Zhang, Yizhi Zhang, Tsz Yui Zhuang, Leqi Wu, Yan Wang, Rongrong Ren, Lai Jiang, Xianting Ding

**Affiliations:** ^1^ Department of Anesthesiology and Surgical Intensive Care Unit Xinhua Hospital School of Medicine and School of Biomedical Engineering Shanghai Jiao Tong University Shanghai 200030 P. R. China; ^2^ State Key Laboratory of Oncogenes and Related Genes Institute for Personalized Medicine Shanghai Jiao Tong University Shanghai 200030 P. R. China; ^3^ Department of Anesthesiology and Surgical Intensive Care Unit Xinhua Hospital Shanghai Jiaotong University School of Medicine Shanghai 200092 P. R. China

**Keywords:** cerebral organoids, exosomes, microenvironment, microfluidic platform, proteomics

## Abstract

Proteomic communications in neighboring microenvironments during early organ development is a dynamic process that continuously reshapes human embryonic stem cells (hESCs) developmental fate. Such dynamic proteomic alteration in the microenvironment consists of both freely secreted proteome and exosome‐encapsulated proteome. Simultaneous monitoring of the time‐lapse shift of both proteomes with live organoids remains technically challenging. Here, a continuous organoid secretion/encapsulation proteome tandem LC‐MS/MS (COSEP‐LCM) is introduced, which permits time‐lapse monitoring of proteomic alterations both in free secretion form and in exosome encapsulated form at live organoids’ microenvironment. Continuous growth of human cerebral organoids (COs) and free‐secretion/exosome‐encapsulation proteomics acquisition with COSEP‐LCM for 60 days is demonstrated. SERPINF1, F5, and EFNB1 are initially enriched inside exosomes as encapsulated excretion and then gradually enriched outside exosomes as freely secreted excretion, while C3 is initially enriched outside exosomes as freely secreted excretion and gradually enriched inside exosomes as encapsulated excretion. Such dynamic excretion pattern paradigm shift may imply critical developmental strategy evolution during early human cerebral development. COSEP‐LCM offers a platform technique for continuous inside/outside exosome proteomics co‐analysis in live organoids’ microenvironment.

## Graphical abstract

Proteomic communications in neighboring microenvironments during early organ development is a dynamic process continuously reshaping hESCs developmental fate. Such dynamic proteomic alteration consists of freely secreted proteome and exosome‐encapsulated proteome. Here, a continuous organoid secretion/encapsulation proteome tandem LC‐MS/MS (COSEP‐LCM) is introduced, permitting time‐lapse monitoring of proteomic alterations in free secretion form and exosome encapsulated form at live organoids’ microenvironment.

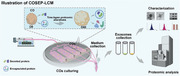

## 1 Introduction

The cell microenvironment is an intricate system of several cell types, including endothelial cells, fibroblast cells, and immune cells, which are recognized for their ability to secrete a wide range of proteins, including cytokines, growth factors, and proteinases.^[^ ^1^ ^]^ The complex interactions between these excreted proteins and receptors on the cell surface are crucial for intracellular communication.^[^ ^2^ ^]^ These protein‐mediated cellular communication and interactions play a crucial role in determining the developmental fate of human embryonic stem cells (hESCs) during early organ development. Alteration in these processes can contribute to the development and progression of central nerve diseases.^[^ ^3^ ^]^ These years, hESCs and hESC‐derived cerebral organoids (COs) have proven to be highly beneficial in human disease modeling^[^ ^4^ ^]^ and innovative treatments via protein‐mediated cellular communication,^[^ ^5^ ^]^ because of the inherent species variations between human beings and animals that restrict their wide application in revealing the mechanism of human specified diseases.^[^ ^6^ ^]^ These secreted proteins in the live COs microenvironment include exosome‐encapsulated proteomes and freely‐secreted proteomes.

The COs possess high therapy potential, while their direct application in medical interventions is limited by concerns regarding safety and ethical issues.^[^ ^7^ ^]^ Paracrine secretion, specifically through the release of exosomes, is one of the primary mechanisms of hESCs and COs for their therapeutic effects,^[^ ^8^ ^]^ which have gained significant interest due to their crucial roles in cell‐cell communication and regenerative medicine.^[^ ^9^ ^]^ Exosomes are tiny lipid bilayer vesicles with a diameter of 30–200 nm, actively secreted by diverse cells, and widely found in the supernatant of cell cultures or bodily fluids.^[^ ^10^ ^]^ Exosomes play a crucial role in intercellular communication by selectively carrying a variety of bioactive substances, including proteins, enzymes, lipids, and nucleic acids, to specific targets throughout the body.^[^ ^11^ ^]^

Nevertheless, certain proteins, including growth factors and cytokines, may not be consistently encapsulated within these secretory vesicles and thus may be disregarded in investigations that concentrate on these proteins.^[^ ^12^ ^]^ The analysis of both the freely secreted proteome and exosome encapsulated proteome provides a more holistic understanding of how cellular communication influences certain diseases and a more comprehensive treatment strategy.^[^ ^13^ ^]^ Therefore, there is a persistent and unfulfilled demand to simultaneously monitor the time‐lapse shift of both freely secreted proteome and exosome‐encapsulated proteome with live COs. Also, multiple studies have shown the potential application of exosomes derived from stem cells in the treatment of human diseases,^[^ ^14^ ^]^ but very little is known about the biogenesis of COs‐Exos.

In this study, we innovatively constructed a continuous organoid secretion/encapsulation proteome tandem LC‐MS/MS (COSEP‐LCM). We cultured COs and collected culture media for both freely secreted proteomics analysis and exosome‐encapsulated proteomics analysis in five different periods (D0‐4, D4‐10, D10‐14, D14‐34, and D34‐60) through COSEP‐LCM. We isolated exosomes by ultracentrifugation, and the characteristic was evaluated with transmission electron microscopy (TEM), nanoparticle tracking analysis (NTA), and western blotting.^[^ ^15^ ^]^ COSEP‐LCM enables real‐time monitoring of proteomic modifications, both in the form of freely secreted proteins and proteins encapsulated in exosomes, at the microenvironment of live COs. We found that SERPINF1, F5, and EFNB1 were initially concentrated within the inside of exosomes as encapsulated excretion and subsequently concentrated within the outside of exosomes as freely secreted excretion. In contrast, C3 was initially freely secreted protein and gradually concentrated as encapsulated excretion. COSEP‐LCM has the potential to continuously co‐analyze inside/outside exosome proteomics in live organoids’ microenvironment, and the study will be helpful to offer innovative perspectives for future research on exosomes separated from COs, as well as for the selection of treatments.

## 2 Results and Discussions

### 2.1 Establishment of the COSEP‐LCM

**Figure 1:**
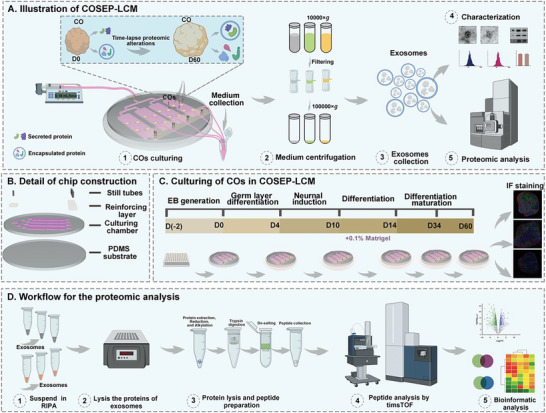
Establishment of the COSEP‐LCM and proteomic analysis. A) Illustration of the COSEP‐LCM. The details of COSEP‐LCM construction and workflow were illustrated. B) Detail of the chip construction. C) Culturing of COs in COSEP‐LCM. Schematic illustration of the main steps in COs generation and culturing protocol. D) Workflow for the proteomic analysis.

To better perform the proteomics analysis of the exosomes derived from COs, and dynamic transformation analysis of the inside/outside proteins secreted from COs exosomes, we constructed a continuous organoid secretion/encapsulation proteome tandem LC‐MS/MS (COSEP‐LCM) (**Figure** **1**A). The COs culture microfluidic platform was adjusted based on our previous work^[^ ^16^ ^]^ (Figure 1B). The details of the chip construction were shown in Figure S1A (Supporting Information). In the current work, the COSEP‐LCM could culture 16 organoids at a time. The organoids were generated according to the reported literature and assembled into the microfluidic chip on the 3rd day of formation (Figure 1C). The initial flow rate used to supply fresh culturing medium to the chip was set at 180 µL h^−1^ and gradually raised by 30 µL h^−1^ every 3 days (Figure S1B, Supporting Information). The culturing media used at different time points were injected according to the literature. The waste media flow through the outlets was collected at different periods (D0‐4, D4‐10, D10‐14, D14‐34, and D34‐60) to isolate exosomes. Then, these exosomes and culture medium from different stages were used for proteomic analysis and dynamic transformation analysis of the inside/outside proteins (Figure 1D).

### 2.2 Characterization of hESCs and COs

**Figure 2:**
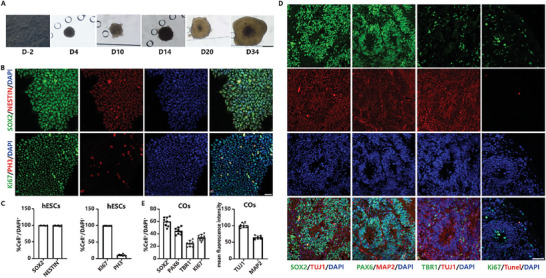
Characterization of the COs. A) Representative brightfield microscope images of the COs at various time points. The scale bar is 500 µm. B) Immunofluorescence staining images of the hESCs for SOX2, NESTIN, Ki67, and PH3. The scale bar is 20 µm. C) Quantification of the percentage of SOX2^+^, NESTIN^+^, Ki67^+^, and PH3^+^ cells to DAPI^+^ cells in hESCs (n = 8). D) Immunofluorescence staining images of the COs cultured under the 3D multichannel microfluidic platform. The scale bar is 20 µm.

Organoids were cultured in the COSEP‐LCM for 60 days and collected for immunofluorescence staining to evaluate the properties of the organoids. The culturing of COs replicated the chronological progression of human brain development by incorporating all the different types of neural cells naturally present in vivo.^[^ ^17^ ^]^ The morphological changes of the organoids were captured through bright field microscope at various time intervals (D4, 10, 14, 20, 34) (**Figure** **2**A). To evaluate the properties of the hESC, the stem cell markers (neuroepithelial stem cell protein (Nestin) and SRY‐box transcription factor 2 (SOX2)), and proliferation markers (Ki67 and Phosphohistone H3 (PH3))^[^ ^18^ ^]^ were stained in hESCs (Figure 2B,C). The results demonstrated that the hESC used to generate COs has proliferating and multipotential differentiation properties. Furthermore, at D34, COs were immunofluorescence stained for stem cell markers (SOX2), various neural markers (MAP2B, TUJ1, and PAX6), proliferation markers (Ki67), apoptosis marker (TUNEL) and preplate marker (TBR1)^[^ ^19^ ^]^ (Figure 2D; Figure S2, Supporting Information) to characterize the COs. In addition, at D60, COs were immunofluorescence stained for cortical layer marker (CTIP2), and astrocyte markers (GFAP) (Figure S3, Supporting Information). These results indicated that our COs model could accurately recapitulate the basic characteristics of the human brain.

### 2.3 Exosomes Isolation and Identification

**Figure 3:**
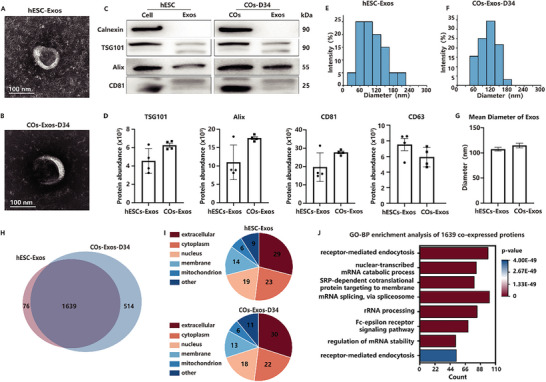
Evaluation of the isolated exosomes. A) Representative TEM image of exosomes derived from hESCs. The scale bar is 100 nm. B) Representative TEM image of exosomes derived from COs on D34. The scale bar is 100 nm. C) Western blotting analysis detecting the expression of Calnexin, TSG101, Alix, and CD81 in cells, COs, and exosomes. D) Protein abundance of TSG101, Alix, and CD81. E) Exosomes derived from hESCs size distribution as determined by NTA. F) Exosomes derived from COs on D34 size distribution as determined by NTA. G) The mean diameter of exosomes derived from hESCs and COs on D34. H) Venn diagram of COs‐Exos on D34 versus hESCs‐Exos. I) The cytolocalization of proteins of hESCs‐Exos and COs‐Exos on D34. J) GO‐BP analysis of the 1639 co‐expressed proteins.

Exosomes were extracted from the culture medium of hESCs and COs on D34 and characterized using morphology, particle size, and surface markers. In this study, we used ultracentrifugation to extract exosomes. Compared with other emerging methods based on nanoscience for exosome separation, ultracentrifugation has the advantages of simple operation, no need for special reagents, and suitability for large‐scale samples.^[^ ^20^ ^]^ TEM analysis revealed the presence of vesicles with a characteristic cup‐shaped morphology (**Figure** **3**A,B). No discernible variations in shape were identified between the exosomes derived from the hESCs and COs. The Western blot analysis revealed that the positive markers, TSG101, Alix, and CD81, were present in the isolated exosomes, but the negative marker, Calnexin, was absent^[^ ^21^ ^]^ (Figure 3C). The results indicated that the separation process was efficient and the exosomes were pure for proteomics analysis. The protein abundance of TSG101, Alix, and CD81 were analyzed (Figure 3D). The particle size distribution of exosomes ranged from 30 to 240 nm, with the majority of exosomes having a diameter of 110 nm (Figure 3E,F). The mean diameter of the exosomes derived from the hESCs and COs is 105 and 111.56 nm, respectively (Figure 3G). The concentration of the exosomes derived from the hESCs and COs is 1.2 × 10^7^ and 1.4 × 10^7^ mL^−1^.

To further characterize the COs exosomes (COs‐Exos) on D34, we conducted proteomic analysis and utilized hESCs exosomes (hESCs‐Exos) as a control for comparison. A total of 2229 proteins were discovered, with ≈73.5% (1639/2229) of the proteins overlapping (Figure 3H). Then, we characterized the abundance curve of the two exosome types (Figures S4, S5A, Supporting Information). Approximately 80% of proteins found in both types of exosomes were consistent with ExoCarta (a comprehensive database of exosome markers), suggesting a high level of reliability in the data (Figure S5B,C, Supporting Information). Then, we analyzed the 1715 proteins expressed in the hESCs‐Exos and the 2153 proteins expressed in the COs‐Exos, respectively. The DAVID website was used to conduct GO analysis and KEGG pathway analysis. According to the results of GO analysis for cellular component terms, the proteins expressed in the hESCs‐Exos were mainly located in the extracellular region, followed by the cytosol. And the proteins expressed in COs‐Exos on D34 had a similar cellular component (Figure 3I). Then, we conducted GO‐BP enrichment analysis on the 1639 proteins that were co‐expressed in hESCs‐Exos and COs‐Exos (Figure 3J). The results indicated that there was no essential difference between the COs‐Exos on D34 and hESCs‐Exos in terms of structural proteins.

### 2.4 Proteomic Analysis of Exosomes from COs in Five Different Periods

**Figure 4:**
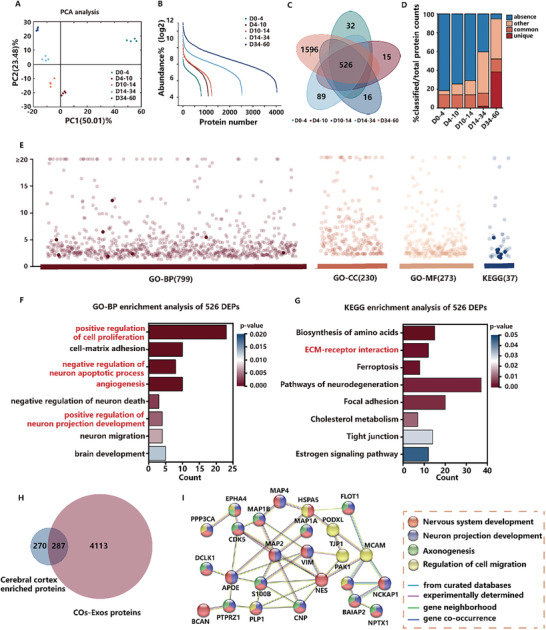
Bioinformatics analysis of COs‐Exos in five different periods. A) Principal component analysis (PCA) of protein relative abundance attained from proteomics data of the COs‐Exos in five different periods. B) Expression abundance curve of the four exosome types. C) Venn diagram of COs‐Exos in five different periods. D) A bar chart illustrating the percentage of classified proteins in five different periods. E) GO and KEGG analysis of COs‐Exos for 526 commonly expressed proteins. F) GO‐BP enrichment analysis of COs‐Exos for 526 commonly expressed proteins. G) KEGG enrichment analysis of COs‐Exos for 526 commonly expressed proteins. H) Venn diagram of COs‐Exos proteins versus cerebral cortex enriched proteins. I) Protein‐protein interaction network reveals the interaction between the 287 proteins related to nervous system development, neuron projection development, axonogenesis, and regulation of cell migration. The color of the nodes represents different pathways. The color of the lines represents the protein‐protein association.

To get a comprehensive protein profile of the COs‐Exos at various phases in COSEP‐LCM, we collected the organoids medium to isolate exosomes in different periods, including D0‐4, D4‐10, D10‐14, D14‐34, and D34‐60 for proteomic analysis. The time periods for medium collection were chosen based on the key steps of COs development, which include embryoid body formation (D0‐4), neural induction (D4‐10), neural differentiation (D10‐14), early maturation (D14‐34), and late maturation (D34‐60).^[^ ^22^ ^]^ It is reported that COs at D60 will differentiate into more cell types, such as glial cells and mature neurons.^[^ ^23^ ^]^ A total of 4027 proteins were detected from the COs‐Exos in five different periods. The proteins that had a p‐value less than 0.05 and a foldchange (FC) more than 1.5 or less than 0.667 were classified as differentially expressed proteins (DEPs). The PCA plot analysis shows that there are significant differences between exosomes in the five different steps of COs development, and there is consistency between exosomes at the same stage of COs development (**Figure** **4**A). Then, we characterized the abundance curve of the four exosomes (Figure 4B). Across the five different periods, the total proteins were analyzed by Venn diagram. 526 proteins were commonly expressed in the five different periods, 32 proteins were specifically expressed during D0‐4, 15 proteins were specifically expressed during D4‐10, 16 proteins were specifically expressed during D10‐14, 63 proteins were specifically expressed during D14‐34, and 1925 proteins were specifically expressed during D34‐60 (Figure 4C,D).

Then, we analyzed these 526 commonly expressed proteins (Figure 4E). Through GO analysis, the 526 shared proteins related to positive regulation of cell proliferation, cell‐matrix adhesion, negative regulation of neuron apoptotic process, angiogenesis, and positive regulation of neuron projection development were identified in the biological process (Figure 4F). Moreover, proteins were shown to be enriched in the extracellular exosome at the cellular component level and enriched in cadherin binding involved in cell‐cell adhesion at the molecular function level (Figure S6, Supporting Information). In addition, the KEGG pathway analysis revealed that the shared proteins were considerably enriched in the biosynthesis of amino acids, ECM‐receptor interaction, and focal adhesion (Figure 4G).

The proteins derived from COs‐Exos exhibited signature overlaps with the cerebral cortex‐enriched proteomic database of Human Protein ATLAS (Figure 4H) (Table S3, Supporting Information). The string analysis revealed that these 287 overlapped proteins were associated with nervous system development, neuron projection development, axonogenesis, and regulation of cell migration (Figure 4I). We found some solute carrier family (SLC1A3, SLC2A3, SLC3A2, and SLC16A1), ATPase family (ATP1A3, ATP2B1, ATP2B4, ATP5F1A, ATP6V0A1, and ATP6V1B2) were listed among the highly expressed list of COs‐Exos. SLC1A3 encodes glutamate transporter, and EAAT1 is the primary and most prevalent transporter responsible for the elimination of the neurotransmitter glutamate in the brain.^[^ ^24^ ^]^ Humans lacking EAAT1 are linked to episodic ataxia,^[^ ^25^ ^]^ and in mice, mutations in EAAT1 lead to a decrease in motor coordination and an increased vulnerability to cerebellar damage.^[^ ^26^ ^]^ The Na^+^/K^+^‐ATPase (NKA) is a ubiquitously expressed transmembrane enzyme that facilitates the active transport of Na^+^ and K^+^ ions by ATP energy across the plasma membranes and is composed of subunits α, β, and occasionally regulatory γ.^[^ ^27^ ^]^ ATP1A3, encoding the α3‐subunit of NKA, is crucial in modulating neuronal excitability and knockout of ATP1A3 associated with human congenital hydrocephalus and perinatal seizure.^[^ ^27^ ^]^ Furthermore, NESTIN, essential for survival, regeneration, and mitogen‐stimulated proliferation of neural progenitor cells,^[^ ^28^ ^]^ and MAP2 and MAP1B, key members in the neuronal microtubule‐associated proteins,^[^ ^29^ ^]^ were also listed among the highly expressed list of hESCs‐Exos. In conclusion, the 287 overlapped proteins have the potential to be utilized as therapeutic agents for human brain disorders.

**Figure 5:**
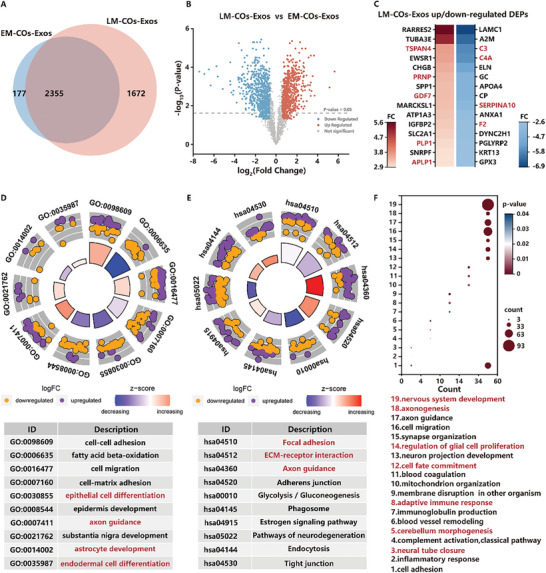
Proteomic comparison of EM‐COs‐Exos and LM‐COs‐Exos. A) Venn diagram of Exos in early mature COs and late mature COs. B) Volcano plot of LM‐COs‐Exos versus EM‐COs‐Exos. C) LM‐COs‐Exos up/down‐regulated DEPs. D) GO‐BP enrichment analysis of LM‐COs‐Exos versus EM‐COs‐Exos. E) KEGG enrichment analysis of LM‐COs‐Exos versus EM‐COs‐Exos. F) GO‐BP enrichment analysis of COs‐Exos in five different periods.

Then, we compared the Exos secreted from early mature COs (EM‐COs‐Exos) and late mature COs (LM‐COs‐Exos). The Venn Diagram and Volcano plot showed that there were 2355 common DEPs between the EM‐COs‐Exos group and LM‐COs‐Exos group (**Figure** **5**A,B). Heat map plots showed the top 15 up/down‐regulated DEPs identified in the LM‐COs‐Exos group (Figure 5C). Then, GO analysis was performed to further explore the difference between the LM‐COs‐Exos group and EM‐COs‐Exos group. As Figure 5D shows, the DEPs in the LM‐COs‐Exos group were primarily enriched in epithelial cell differentiation, axon guidance, astrocyte development, and endodermal cell differentiation, which means that compared to the EM COs Exos group, LM‐COs‐Exos group was associated with differentiation of different cell types. In addition, we performed KEGG analysis, and the results enriched in Focal adhesion, ECM‐receptor interaction, and Axon guidance (Figure 5E).

Lastly, we analyzed the proteins that are uniquely and highly expressed in each group, including D0‐4 group, D4‐10 group, D10‐14 group, D14‐34 group, and D34‐60 group, respectively. GO analysis on the uniquely and highly expressed proteins in each group indicated that the D0‐4 proteins were mainly enriched in neural tube closure and inflammatory response, the D4‐10 proteins were primarily enriched in cerebellum morphogenesis and blood vessel remodeling, the D10‐14 proteins were largely enriched in membrane disruption in other organism and adaptive immune response, the D14‐34 proteins were significantly enriched in cell fate commitment and mitochondrion organization, and the D34‐60 proteins were mainly enriched in regulation of glial cell proliferation, synapse organization, cell migration, axon guidance, axonogenesis, and nervous system development (Figure 5F). Compared to other stages, the proteins from D34‐60, namely the late maturation step, are more related to glial cell proliferation and nervous system development. In contrast, the proteins from D0‐4, D4‐10, and D10‐14, namely embryoid body formation, neural induction, and neural differentiation step, are more related to the complement system and immune response. In addition, we performed KEGG analysis on the proteins that are uniquely expressed in the D34‐60 group. The results enriched in Endocytosis, Axon guidance, Focal adhesion, mTOR signaling pathway, Wnt signaling pathway, and Longevity regulating pathway (Figure S7, Supporting Information).

### 2.5 Alteration of Freely Secreted Proteome and Exosome Encapsulated Proteome from COs in Five Different Periods

**Figure 6:**
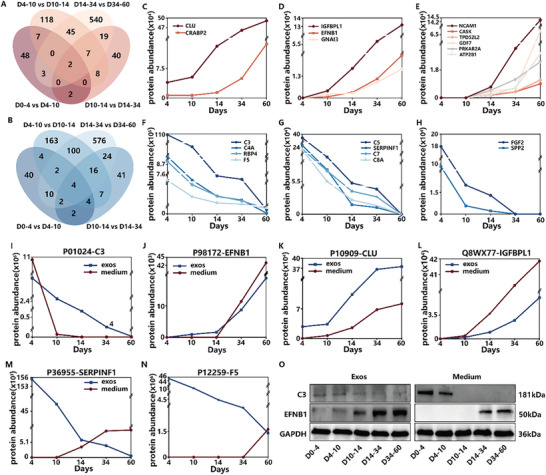
Bioinformatics analysis of COs‐Exos inside/outside proteins. A) Venn diagram of upregulated proteins in COs‐Exos. B) Venn diagram of downregulated proteins in COs‐Exos. C) Line graph of the upregulated proteins in COs‐Exos. D) Line graph of the upregulated proteins in COs‐Exos, including proteins with an expression level of 0 on D0‐4. E) Line graph of the upregulated proteins in COs‐Exos, including proteins with an expression level of 0 on D0‐4 and D4‐10. F) Line graph of the downregulated proteins in COs‐Exos. G) Line graph of the downregulated proteins in COs‐Exos, including proteins with an expression level of 0 on D0‐4. H) Line graph of the downregulated proteins in COs‐Exos, including proteins with an expression level of 0 on D0‐4 and D4‐10. I–N) Inside/outside differentially expressed proteins in COs‐Exos. O) Western blot images for C3, EFNB1, and GAPDH in Exos and medium.

We compared the proteins in the D0‐4 group with the D4‐10 group, the D4‐10 group with the D10‐14 group, the D10‐14 group with the D14‐34 group, and the D14‐34 group with the D34‐60 group (**Figure** **6**A,B; Figure S8, Supporting Information). We found there were two proteins, CLU and CRABP2, consistently upregulated in expression from D0 to D60 (Figure 6C). When including proteins with an expression level of 0 on D0‐4, there were other three proteins, EFNB1, GANAB, and IGFBPL1, consistently upregulated in expression from D0 to D60 (Figure 6D). When including proteins with an expression level of 0 on D0‐4 and D4‐10, there were other six proteins, NCAM1, CASK, TPD52L2, GDF7, PRKAR2A, and ATP2B1, consistently upregulated in expression from D0 to D60 (Figure 6E). Moreover, there were four proteins, C3, C4A, F5, and RBP4 consistently downregulated in expression from D0 to D60 (Figure 6F). When including proteins with an expression level of 0 on D34‐60, there were other four proteins, C5, SERPINF1, C7, and C8A, consistently downregulated in expression from D0 to D60 (Figure 6G). In addition, when including proteins with an expression level of 0 on D14‐34 and D34‐60, there were other two proteins, FGF2 and SPP2, consistently downregulated in expression from D0 to D60 (Figure 6H). The majority of the down‐regulated proteins, such as C3, C5, C4A, and C7, belong to the complement system. It was reported that complement‐related proteins could modulate synapse activity in the cortex, hence influencing brain development.^[^ ^30^ ^]^

Then, we compared the inside proteins secreted as exosome‐encapsulation and the outside proteins secreted as free‐excretion, which excluded the exosomes and the proteins inherent in the culture medium. The inside proteins and the outside proteins were analyzed in a line graph. Our analysis revealed that the expression of C3 in both inside proteins and outside proteins during D0‐60 decreased consistently (Figure 6I). The expression of EFNB1, CLU, and IGFBPL1 in both inside proteins and outside proteins during D0‐60 increased consistently (Figure 6J–L). Moreover, the SERPINF1 and F5 expressed in exosomes were reduced in inside proteins and increased in outside proteins during COs culture (Figure 6M,N). Meanwhile, EFNB1, F5, and SERPINF1 were initially enriched in inside proteins and then gradually enriched in outside proteins, while C3 was initially enriched in outside proteins and gradually enriched in inside proteins. Then, we used Western blot to identify the expression changes of C3 and EFNB1 in inside proteins and outside proteins (Figure 6O). The results of Western blot are consistent with those of proteomics. C3 and CLU are complement proteins, playing a critical role in innate immunity. The expression of C3 and CLU has been linked to the generation or clearance of Abeta, which is associated with the pathogenesis of Alzheimer's disease.^[^ ^31^ ^]^ EFNB1 belongs to the family of membrane‐linked signaling molecules known as Eph/ephrin and is crucial for cell migration, adhesion, axon guidance, neurogenesis, and vascular development.^[^ ^32^ ^]^ IGFBPL1, expressed in human microglia, could regulate the growth of retinal ganglion cell axons,^[^ ^33^ ^]^ enhance cognitive functions through modulating striatal neuronal activity,^[^ ^34^ ^]^ and mitigate neuroinflammation to prevent neurodegenerative disease.^[^ ^35^ ^]^ SERPINF1, a 50‐kDa secreted glycoprotein, has neuroprotective, antiangiogenic, and antitumorigenic effects.^[^ ^36^ ^]^ The level of SERPINF1 exhibits a correlation with some neurological disorders, such as traumatic brain injury, cerebral ischemia, and Alzheimer's disease. In addition, elevated SERPINF1 levels in the cerebrospinal fluid have been suggested as a potential biomarker for Alzheimer's disease.^[^ ^37^ ^]^ F5, the central regulator of hemostasis, is reported to be correlated with genetic defects of thrombophilia in childhood porencephaly.^[^ ^38^ ^]^ Such dynamic excretion pattern paradigm shift may imply critical developmental strategy evolution during early human cerebral development and offer comprehensive alternative treatment strategies in central nervous system diseases.

## 3 Conclusion

In this work, we constructed COSEP‐LCM, which permits the dynamic transformation analysis of the free‐secretion/exosome‐encapsulation proteins secreted from COs‐Exos for 60 days. Our studies revealed that early COs‐Exos (D0‐34) were shown to be linked to immune response, whereas LM‐COs‐Exos (D34‐60) were observed to have a greater impact on brain development and differentiation of different cell types. The results of our study will offer valuable insights for choosing the most suitable source for exosomes, to enhance research and therapeutic effectiveness. Moreover, through the COSEP‐LCM, we found that during the process of COs culturing, there were three proteins (EFNB1, CLU, IGFBPL1) in both freely secreted proteome and exosome encapsulated proteome increased consistently, while one protein (C3) decreased consistently. Moreover, EFNB1, F5, and SERPINF1 were initially enriched inside protein (exosome) and then gradually enriched outside protein (freely secretion), while C3 underwent a reverse biological process. In conclusion, COSEP‐LCM provides a comprehensive approach for the analysis of the protein composition of exosomes derived from COs, including protein distribution at different stages and continuous inside/outside exosome proteomics co‐analysis in live organoids’ microenvironment.

## 4 Experimental Section

### Culture and Identification of hESCs

The H9 human embryonic stem cell (H9‐hESC) line was provided as a gift by Dr. Zhenge Luo. Additionally, the mycoplasma test result was negative. Then, a six‐well plate (Corning) was pre‐coated with hESC‐qualified Matrigel (Corning). The hESCs were grown in mTeSR Plus medium (STEMCELL) on the six‐well plate, and 2 mL of new culture medium was replaced daily.

### Generation and Differentiation of the Cerebral Organoids

The cerebral organoids were generated and differentiated based on previous research^[^ ^22^, ^39^ ^]^ after some modifications. First, a V‐bottom 96‐well plate (Thermo) was treated with lipidure (NOF CORPORATION) and sterilized overnight by UV radiation. After the confluence of hESCs reached ≈80–90%, the cells were dissociated into single cells with Accutase (STEMCELL) at a temperature of 37 °C for 4–5 min, and were resuspended in StemFlex medium (Gibco) added with 10 µm Y27632 (STEMCELL). Then, these cells were plated into the V‐bottom 96‐well plate treated above with 9000 cells/well, 150 µL well^−1^ for embryoid bodies (EBs) formation. The EBs were transferred to the chip after formation (D0) and the ectodermal induction medium containing 80% (v/v) DMEM/F12 (Life/Invitrogen), 20% (v/v) Knockout Serum Replacer (Gibco), 3.5 µL L^−1^ β‐mercaptoethanol (Sigma–Aldrich), 2.5 µm dorsomorphine (Tocris), 2 µm A83‐01 (Tocris), 1% (v/v) MEM‐NEAA (Gibco), and 1% (v/v) Glutamax (Gibco) to promote germ layer differentiation. On D4, the medium was changed to neural induction medium containing 97% (v/v) DMEM/F12, 1% (v/v) MEM‐NEAA, 1% (v/v) Glutamax, 1% (v/v) N2 supplement (Life/Invitrogen), 10 µm SB431542 (Selleck), 1 µg mL^−1^ heparin (Sigma–Aldrich), and 200 nm LDN193189 2HCL (Selleck) to induce the neural ectoderm. On D10, the medium was changed to differentiation medium containing 50% (v/v) DMEM/F12, 50% (v/v) Neurobasal medium (Life/Invitrogen), 0.5% (v/v) B27 supplement without vitamin A (Life/Invitrogen), 0.5% (v/v) N2 supplement, 250 µL L^−1^ Insulin (Sigma–Aldrich), 3.5 µL L^−1^ β‐mercaptoethanol, 0.5% (v/v) MEM‐NEAA, 1% (v/v) Glutamax, and 1% (v/v) Antibiotic‐Antimycotic (Gibco). On D14, the B27 supplement without vitamin A was substituted with the B27 supplement (Life/Invitrogen) with vitamin A during the maturation process. The reagents and chemicals used in this work are included in Table S1 (Supporting Information).

### Chip Design

The microfluidic chip applied for culturing cerebral organoids and collecting exosomes used in this work was modified from our previous work. The microfluidic chip had one inlet, four outlets, and four chambers with four separate capturing regions. Each capturing region had multiple pillars with a diameter of 300 µm to hold the organoids in the region. The usage of the pillars was to hold the organoids in the chip, prevent being flushed away during the culturing process, and prevent the organoids from sticking together. Briefly, the width of the channel for organoid injection was 800 µm. The width of the chamber was 9100 µm, the length of the chamber was 31 mm, and the diameter of the capturing region was 3.7 mm. The height of the chip was 500 µm. The parameters were optimized based on previous work.

### Chip Fabrication

The silicon wafer with a designed pattern to make the chip was fabricated according to our previous work. Briefly, the 2D draft of the chip was designed by AutoCAD software, and the photomask of the draft was printed by a company. To achieve a wafer with a height of 500 µm, the SU‐8 2100 was uniformly spun coated on a clean 3‐inch silicon wafer, baked on the hot plate, exposed to the UV light, post baked on the hot plate at 65 and 95 °C for 10 min and 2 h, respectively. Then, the pattern on the wafer was developed with the developer. The wafer was placed on a hot plate for 1 h at 120 °C and salinized with Chlorotrimethylsilane for 5 min to prevent the pattern from tearing off from the wafer. Then the degassed PDMS mixed with a curing agent (Sylgard 184, DOW CORNING, USA) at an 11:1 ratio was poured on the wafer, vacuumed, and baked on a hotplate at 85 °C for 45 min. The substrate for chip bonding was also made by PDMS. The solidified PDMS was peeled off, cut into shapes, punched holes in the inlet and outlets, cleaned with tape, and bonded to the PDMS substrate via oxygen plasma (Harrick plasma, USA). Steel pipes with a diameter of 0.8 mm were inserted into the inlet and outlets. Then, the chip surface was modified with 0.5 wt% Lipidure‐CM ethanol solution for 30 min to make the chip surface more suitable for organoid growth.

### Microfluidic Assembly of Cerebral Organoids

The cerebral organoids were injected into the microfluidic chip by a 1 mL syringe after being cultured in mTeSR1 medium for 2 days. Briefly, the chip was washed with hESC medium to remove the bubbles in the chip before injecting the organoid. The flexible Tygon tubes with an inner diameter of 0.8 mm and 1 mL syringes with hESC medium were connected to each outlet. During the injection of the organoids into the chip process, the syringe connected to the outlet was removed to allow the organoids to flow to the corresponding chamber according to the pressure difference between the inlet and outlet. The positions of the organoids in the chamber were controlled by adjusting the chip position and allowing the organoids to settle down in different capturing regions.

### Isolation of Exosomes

The isolation of exosomes was conducted by ultracentrifugation, in accordance with a previously described study.^[^ ^14a^ ^]^ Due to the culture medium, all serum‐free hESCs and the cerebral organoids were used, the medium was collected and used for gradient centrifugation at 4000 × g for 10 min, and 10000 × g for 10 min to remove cells and debris. Then, a 0.22 µm pore filter (Sigma) was used to filter the supernatant. The filtered supernatant was gathered and ultracentrifuged at 100000 × g for 90 min at a temperature of 4 °C to preserve the precipitated pellets of exosomes. The precipitated pellets were resuspended in PBS, and subsequently subjected to centrifugation, following the procedure as described above. The final exosomes were resuspended in PBS and kept at a temperature of −80 °C for further examination.

### Characterization of Exosomes

Transmission electron microscopy (TEM) was employed to describe the morphological characteristics of the isolated exosomes. Nanoparticle tracking analysis (NTA) was employed to detect the concentration and size of the isolated exosomes. Western blotting was employed to examine the expression levels of surface markers, such as calnexin (ab13504), Alix(ab117600), CD81 (ab219209), and Tsg101 (ab125011), on exosomes.

### Immunofluorescence Staining

The immunofluorescence staining for the cerebral organoids was performed in accordance with a previously described protocol.^[^ ^40^ ^]^ In short, the cerebral organoids were washed with DPBS (Gibco), fixed in 4% paraformaldehyde (PFA) solution for 6–8 h at a temperature of 4 °C, washed with DPBS again, and finally dehydrated in 30% sucrose solution at a temperature of 4 °C until sunk. The organoids were embedded in the optimal cutting temperature compound (OCT) and sliced at a thickness of 10 µm before immunofluorescence staining. Then these slides were permeabilized in 0.2% Triton X‐100 for 30 min, washed with PBS three times, retrieved with antigen retrieval reagent (Beyotime) for 5 min, washed with PBS for three times again, and blocked with blocking solution (Beyotime) for 1 h. Subsequently, the slides were incubated with primary antibodies overnight and secondary antibodies for 2 h. Finally, DAPI (Sigma–Aldrich) was used to counterstain. All images were acquired utilizing a Zeiss LSM 880 confocal microscope. The primary and secondary antibodies used in the immunofluorescence staining are included in Table S2 (Supporting Information).

### Western Blotting

The Western blotting procedure was performed according to a previously established protocol.^[^ ^40^ ^]^ In short, the cerebral organoids were homogenized, lysed, and centrifuged to prepare the protein sample. The proteins that underwent denaturation, with a total of 30 µg, were loaded onto a 10% SDS‐PAGE gel and separated by electrophoresis. Subsequently, they were electrically transferred to a polyvinylidene fluoride membrane. After the non‐specific binding was blocked with blocking solution at room temperature for 2 h, the primary antibody was reacted in a fridge at 4 °C overnight. Then, the secondary antibody was linked at room temperature for 2 h. The ECL reagent (Yeasen) was used to visualize the protein bands. The following antigens, CALNEXIN, ALIX, CD63, and CD81 were identified. The primary and secondary antibodies used in Western blotting are included in Table S2 (Supporting Information).

### Proteomics Sample Preparation

The isolated exosomes were suspended with 100 µL 50 mm Ammonium bicarbonate (NH₄HCO₃). 100 µL of RIPA solution containing 1 mm PMSF and 100X protease inhibitor cocktail was added to the exosome suspension, and incubated for 10 min on a metal bath pot at 95 °C. Then, the lysates were centrifugated and the supernatants were removed to a new tube, followed by precipitating with −20 °C acetone overnight in a −20 °C fridge. The proteins were collected by centrifuging and discarding the supernatants. Afterward, the proteins were reduced by 1 m dithiothreitol (DTT) and 7 m Guanidine hydrochloride for 1 h at 55 °C, and alkylated by 1 m iodoacetamide (IAA) in 50 mm NH₄HCO₃ solution for 30 min in dark at room temperature. The reduced and alkylated proteins were washed with 50 mm NH₄HCO₃ in 10 kDa ultrafiltration tubes (10 kDa, Sartorius AG) three times and digested with trypsin in an enzyme/protein weight ratio of 1:50 at 37 °C overnight to attain peptides. The harvested peptides were desalted in a Macro Spin Column TARGA C18 (Nest Group) and eluted off with 100 µL 60% acetonitrile (ACN), 40% water, and 0.1% FA. The eluted peptides were vacuum‐dried and saved in −80 °C fridge for use. Before analyzing with the mass spectrum, the peptides were resolved with 0.1% formic acid.

### Mass Spectrometry Analysis

The resuspended peptides were measured for concentration and 10 µL of peptides at the concentration of 1 ng µL^−1^ were added in the autosampler Vials. The peptides were analyzed with the nanoElute timsTOF Pro system (Bruker) following the 90 min LC gradients. For protein identification, the raw files obtained by TimsTOF were searched against the human database (9606‐homo sapiens) by Data‐Independent Acquisition by Neural Networks (DIA‐NN) using the default parameters.

### Bioinformatics Analysis

The data of detected proteins attained from DIA‐NN were used for downstream analyses. The K Nearest Neighbors (KNN) algorithm was used for missing value padding and hypothesis testing was used to acquire the significantly differentiated proteins (DEPs). The DEPs were used for Gene Ontology (GO), and Kyoto Encyclopedia of Genes and Genomes (KEGG) analyses. The GO and KEGG analysis were conducted by DAVID (database for annotation, visualization, and integrated discovery, https://david.ncifcrf.gov/).

### Statistical Analysis

In this study, the sample size (n) for each statistical analysis was 4. The data presented in this study were expressed as the mean ± standard error of the mean (SEM) and analyzed using the GraphPad Prism9 software (GraphPad). The data were subjected to statistical analysis using a one‐way analysis of variance (ANOVA) followed by Tukey's multiple comparisons test to assess the variations among different groups. In this investigation, a level of *P* <0.05 was deemed to be statistically significant.

## Conflict of Interest

The authors declare no conflict of interest.

## Author Contributions

H.Y. and A.A. contributed equally to this work. H.Y. performed conceptualization, methodology, software, data curation, and wrote the original draft. A.A. did formal analysis, methodology, and wrote the original draft and editing. A.W. did formal analysis, software, and wrote the original draft and editing. S.D. performed software and wrote the original draft and editing. M.Z., Y.Z., T.Y.Z., and L.W. did investigation and validation. Y.W. performed formal analysis and wrote the original draft and editing. R.R., L.J., and X.D. did supervision and wrote the original draft and editing.

## Supporting information



Supporting Information

Supplemental Table 1

Supplemental Table 2

## Data Availability

The data that support the findings of this study are available on request from the corresponding author. The data are not publicly available due to privacy or ethical restrictions.
