# Supplementary material for: Time‐Lapse Acquisition of Both Freely Secreted Proteome and Exosome Encapsulated Proteome in Live Organoids’ Microenvironment
Source: Adv Sci (Weinh). 2024 Nov 21;12(2):2406509. doi: 10.1002/advs.202406509 (PMC11727246; doi:10.1002/advs.202406509)
Supplement: Supplementary file 1 — Supporting Information [file ADVS-12-2406509-s003.docx]

***Supplementary information***

**Time-lapse acquisition of both freely secreted proteome and exosome encapsulated proteome in live organoids’ microenvironment**

Haoni Yan ^a, 1^, Aynur Abdulla ^a, b, 1^, Aiting Wang ^a, b^, Shuyu Ding ^a^, Manlin Zhang ^b^, Yizhi Zhang ^b^, Tsz Yui Zhuang ^b^, Leqi Wu ^b^, Yan Wang ^a^, Rongrong Ren ^c, *^, Lai Jiang ^a, **^, Xianting Ding ^a, b, ***^

^a^ Department of Anesthesiology and Surgical Intensive Care Unit, Xinhua Hospital, School of Medicine and School of Biomedical Engineering, Shanghai Jiao Tong University, Shanghai 200030, People’s Republic of China

^b^ State Key Laboratory of Oncogenes and Related Genes, Institute for Personalized Medicine, Shanghai Jiao Tong University, Shanghai 200030, People’s Republic of China

^c^ Department of Anesthesiology and Surgical Intensive Care Unit, Xinhua Hospital, Shanghai Jiaotong University School of Medicine, Shanghai 200092

* Corresponding authors: Rongrong Ren, Lai Jiang, and Xianting Ding

^1^ Haoni Yan and Aynur Abdulla contributed equally

E-mail: [renrongrong@xinhuamed.com.cn](mailto:renrongrong@xinhuamed.com.cn), [jianglai@xinhuamed.com.cn](mailto:jianglai@xinhuamed.com.cn), [dingxianting@sjtu.edu.cn](mailto:dingxianting@sjtu.edu.cn)

**This PDF file includes:**

Tables S1 to S3

Figs. S1 to S5

**Table S1. Antibodies used in this work.**

| Antibodies | Source | Identifier | Additional information |
| --- | --- | --- | --- |
| Pax6 | Proteintech | Cat# 12323-1-AP | IF (1:500) |
| Sox2 | Millipore | Cat# AB5603 | IF (1:200) |
| Ph3 | Abcam | Cat# ab14955 | IF (1:1000) |
| Ki67 | Proteintech | Cat# 27309-1-AP | IF (1:500) |
| Tbr1 | Proteintech | Cat# 20932-1-AP | IF (1:300) |
| Tuj1 | Proteintech | Cat# 66375-1-Ig | IF (1:500) |
| Map2 | Proteintech | Cat# 67015-1-Ig | IF (1:500) |

**Table S2. Reagents and chemicals used in this work.**

| Chemicals, Peptides and Recombinant Proteins | Source | Identifier |
| --- | --- | --- |
| Neural induction medium (NIM) | STEMCELL | Cat# 05835 |
| Insulin | Sigma-Aldrich | Cat# I9278 |
| Y27632 | STEMCELL | Cat# 72304 |
| Dorsomorphine | Tocris | Cat# 3093/10 |
| LDN-193189 2HCL | Selleck | Cat# S7507 |
| SB431542 | Selleck | Cat# S1067 |
| Lipidure | NOF CORPORATION | Cat# CM5206 |
| Antibiotic- Antimycotic | Gibco | Cat# 15240096 |
| Matrigel hESC- Qualified Matrix | BD-Biocoat | Cat# 354277 |
| mTeSR1 | STEMCELL | Cat# 85850 |
| ReLeSR | STEMCELL | Cat# 05872 |
| Accutase | STEMCELL | Cat# 07920 |
| DMEM-F12 | Gibco | Cat# 11330032 |
| Neurobasal | Gibco | Cat# 21103049 |
| β-mercaptoethanol | Sigma-Aldrich | Cat# M3148 |
| Glutamax | Gibco | Cat# 35050061 |
| MEM-NEAA | Gibco | Cat# 11140050 |
| Knockout Serum Replacer | Gibco | Cat# 10828028 |
| DPBS | Life/Invitrogen | Cat# 14190144 |
| N2 supplement | Life/Invitrogen | Cat# 17502048 |
| B27 supplement without vitamin A | Life/Invitrogen | Cat# 12587010 |
| B27 supplement | Life/Invitrogen | Cat# 17504044 |
| Heparin | Sigma-Aldrich | Cat# H3393 |
| Retinol acetate/Retinyl  Acetate | Sigma-Aldrich | Cat# R4632 |
| Tunel | Roche | Cat# 12156792910 |
| O.C.T | Thermo Fisher Scientific | Cat#6502 |
| TritonX-1000 | Sigma-Aldrich | Cat# T8787 |
| AnTIGEN RETRIEVAL SOLUTION | Beyotime | Cat# P0090 |
| Antibody Dilution Solution | Beyotime | Cat# P0103 |
| Blocking solution | Beyotime | Cat# P0260 |
| RIPA lysis solution | Proteintech | Cat# PR20035 |

**Table S3.** **The proteins co-expressed in COs-Exos and cerebral cortex**

| Protien | Gene | Accession Number |
| --- | --- | --- |
| 4-aminobutyrate aminotransferase, mitochondrial | ABAT | P80404 |
| Abl interactor 2 | ABI2 | Q9NYB9 |
| Cytosolic acyl coenzyme A thioester hydrolase | ACOT7 | O00154 |
| Methylmalonate-semialdehyde dehydrogenase, mitochondrial | ALDH6A1 | Q02252 |
| Apolipoprotein D | APOD | P05090 |
| Apolipoprotein E | APOE | P02649 |
| Sodium/potassium-transporting ATPase subunit alpha-3 | ATP1A3 | P13637 |
| Plasma membrane calcium-transporting ATPase 1 | ATP2B1 | P20020 |
| Plasma membrane calcium-transporting ATPase 4 | ATP2B4 | P23634 |
| ATP synthase subunit alpha, mitochondrial | ATP5F1A | P25705 |
| V-type proton ATPase 116 kDa subunit a 1 | ATP6V0A1 | Q93050 |
| V-type proton ATPase subunit B, brain isoform | ATP6V1B2 | P21281 |
| Methylglutaconyl-CoA hydratase, mitochondrial | AUH | Q13825 |
| Brain-specific angiogenesis inhibitor 1-associated protein 2 | BAIAP2 | Q9UQB8 |
| Brain acid soluble protein 1 | BASP1 | P80723 |
| Bcl-2-associated transcription factor 1 | BCLAF1 | Q9NYF8 |
| Myc box-dependent-interacting protein 1 | BIN1 | O00499 |
| Carbonic anhydrase 2 | CA2 | P00918 |
| Calcyclin-binding protein | CACYBP | Q9HB71 |
| Calreticulin | CALR | P27797 |
| Calnexin | CANX | P27824 |
| Calpain-1 catalytic subunit | CAPN1 | P07384 |
| Carbonyl reductase [NADPH] 1 | CBR1 | P16152 |
| Chromobox protein homolog 5 | CBX5 | P45973 |
| Cell cycle and apoptosis regulator protein 2 | CCAR2 | Q8N163 |
| CD99 antigen-like protein 2 | CD99L2 | Q8TCZ2 |
| Centromere protein V | CENPV | Q7Z7K6 |
| Creatine kinase B-type | CKB | P12277 |
| Calsyntenin-1 | CLSTN1 | O94985 |
| Calponin-3 | CNN3 | Q15417 |
| 2',3'-cyclic-nucleotide 3'-phosphodiesterase | CNP | P09543 |
| Contactin-1 | CNTN1 | Q12860 |
| Citrate synthase, mitochondrial | CS | O75390 |
| Cleavage stimulation factor subunit 3 | CSTF3 | Q12996 |
| Cathepsin B | CTSB | P07858 |
| CYFIP-related Rac1 interactor B | CYRIB | Q9NUQ9 |
| Aspartate--tRNA ligase, cytoplasmic | DARS1 | P14868 |
| DAZ-associated protein 1 | DAZAP1 | Q96EP5 |
| Serine/threonine-protein kinase DCLK1 | DCLK1 | O15075 |
| N(G),N(G)-dimethylarginine dimethylaminohydrolase 1 | DDAH1 | O94760 |
| ATP-dependent RNA helicase DDX1 | DDX1 | Q92499 |
| Aspartyl aminopeptidase | DNPEP | Q9ULA0 |
| Enoyl-CoA hydratase, mitochondrial | ECHS1 | P30084 |
| Elongation factor 2 | EEF2 | P13639 |
| Eukaryotic translation initiation factor 3 subunit I | EIF3I | Q13347 |
| Gamma-enolase | ENO2 | P09104 |
| Band 4.1-like protein 1 | EPB41L1 | Q9H4G0 |
| Ephrin type-A receptor 4 | EPHA4 | P54764 |
| Endoplasmic reticulum resident protein 29 | ERP29 | P30040 |
| Protein FAM98B | FAM98B | Q52LJ0 |
| Phenylalanine--tRNA ligase alpha subunit | FARSA | Q9Y285 |
| Phenylalanine--tRNA ligase beta subunit | FARSB | Q9NSD9 |
| Flotillin-1 | FLOT1 | O75955 |
| Fascin | FSCN1 | Q16658 |
| Ferritin light chain | FTL | P02792 |
| Ras GTPase-activating protein-binding protein 2 | G3BP2 | Q9UN86 |
| Neutral alpha-glucosidase AB | GANAB | Q14697 |
| Neuromodulin | GAP43 | P17677 |
| eIF-2-alpha kinase activator GCN1 | GCN1 | Q92616 |
| ARF GTPase-activating protein GIT1 | GIT1 | Q9Y2X7 |
| Gap junction alpha-1 protein | GJA1 | P17302 |
| Glyoxalase domain-containing protein 4 | GLOD4 | Q9HC38 |
| Cytokine-like nuclear factor N-PAC | GLYR1 | Q49A26 |
| Guanine nucleotide-binding protein G(o) subunit alpha | GNAO1 | P09471 |
| Golgin subfamily A member 3 | GOLGA3 | Q08378 |
| Aspartate aminotransferase, mitochondrial | GOT2 | P00505 |
| Gephyrin | GPHN | Q9NQX3 |
| Glycogen [starch] synthase, muscle | GYS1 | P13807 |
| Histone H1.10 | H1-10 | Q92522 |
| Hydroxyacyl-coenzyme A dehydrogenase, mitochondrial | HADH | Q16836 |
| Hepatoma-derived growth factor-related protein 2 | HDGFL2 | Q7Z4V5 |
| High mobility group protein HMG-I/HMG-Y | HMGA1 | P17096 |
| Heterogeneous nuclear ribonucleoprotein A0 | HNRNPA0 | Q13151 |
| Heterogeneous nuclear ribonucleoprotein U-like protein 1 | HNRNPUL1 | Q9BUJ2 |
| Hypoxanthine-guanine phosphoribosyltransferase | HPRT1 | P00492 |
| Hydroxysteroid dehydrogenase-like protein 2 | HSDL2 | Q6YN16 |
| Endoplasmin | HSP90B1 | P14625 |
| Endoplasmic reticulum chaperone BiP | HSPA5 | P11021 |
| Ras GTPase-activating-like protein IQGAP1 | IQGAP1 | P46940 |
| Integral membrane protein 2B | ITM2B | Q9Y287 |
| Lysine--tRNA ligase | KARS1 | Q15046 |
| Importin subunit alpha-3 | KPNA4 | O00629 |
| Glutathione S-transferase LANCL1 | LANCL1 | O43813 |
| Lamin-B2 | LMNB2 | Q03252 |
| Leucine-rich repeat-containing protein 47 | LRRC47 | Q8N1G4 |
| Leucine-rich repeat-containing protein 59 | LRRC59 | Q96AG4 |
| Luc7-like protein 3 | LUC7L3 | O95232 |
| Microtubule-associated protein 1A | MAP1A | P78559 |
| Microtubule-associated protein 1B | MAP1B | P46821 |
| Microtubule-associated protein 2 | MAP2 | P11137 |
| Mitogen-activated protein kinase 1 | MAPK1 | P28482 |
| Microtubule-associated protein RP/EB family member 2 | MAPRE2 | Q15555 |
| Mediator of DNA damage checkpoint protein 1 | MDC1 | Q14676 |
| Malate dehydrogenase, mitochondrial | MDH2 | P40926 |
| DNA mismatch repair protein Msh6 | MSH6 | P52701 |
| NEDD8-activating enzyme E1 regulatory subunit | NAE1 | Q13564 |
| Gamma-soluble NSF attachment protein | NAPG | Q99747 |
| Neural cell adhesion molecule 1 | NCAM1 | P13591 |
| Nck-associated protein 1 | NCKAP1 | Q9Y2A7 |
| Protein NDRG3 | NDRG3 | Q9UGV2 |
| NADH dehydrogenase [ubiquinone] flavoprotein 1, mitochondrial | NDUFV1 | P49821 |
| NADH dehydrogenase [ubiquinone] flavoprotein 2, mitochondrial | NDUFV2 | P19404 |
| Nestin | NES | P48681 |
| Nidogen-2 | NID2 | Q14112 |
| Non-POU domain-containing octamer-binding protein | NONO | Q15233 |
| Vesicle-fusing ATPase | NSF | P46459 |
| Nuclear mitotic apparatus protein 1 | NUMA1 | Q14980 |
| Prolyl 4-hydroxylase subunit alpha-1 | P4HA1 | P13674 |
| Serine/threonine-protein kinase PAK 1 | PAK1 | Q13153 |
| Protein disulfide-isomerase A3 | PDIA3 | P30101 |
| Sister chromatid cohesion protein PDS5 homolog A | PDS5A | Q29RF7 |
| Pyridoxal kinase | PDXK | O00764 |
| Profilin-2 | PFN2 | P35080 |
| D-3-phosphoglycerate dehydrogenase | PHGDH | O43175 |
| Myelin proteolipid protein | PLP1 | P60201 |
| Podocalyxin | PODXL | O00592 |
| Pogo transposable element with ZNF domain | POGZ | Q7Z3K3 |
| Periostin | POSTN | Q15063 |
| Thioredoxin-dependent peroxide reductase, mitochondrial | PRDX3 | P30048 |
| U4/U6 small nuclear ribonucleoprotein Prp4 | PRPF4 | O43172 |
| PC4 and SFRS1-interacting protein | PSIP1 | O75475 |
| 26S proteasome regulatory subunit 4 | PSMC1 | P62191 |
| 26S proteasome regulatory subunit 8 | PSMC5 | P62195 |
| 26S proteasome non-ATPase regulatory subunit 1 | PSMD1 | Q99460 |
| 26S proteasome non-ATPase regulatory subunit 4 | PSMD4 | P55036 |
| Pleiotrophin | PTN | P21246 |
| Receptor-type tyrosine-protein phosphatase zeta | PTPRZ1 | P23471 |
| Glutamine--tRNA ligase | QARS1 | P47897 |
| UV excision repair protein RAD23 homolog B | RAD23B | P54727 |
| RNA-binding protein Raly | RALY | Q9UKM9 |
| 60S ribosomal protein L10 | RPL10 | P27635 |
| 60S ribosomal protein L32 | RPL32 | P62910 |
| 60S ribosomal protein L8 | RPL8 | P62917 |
| 60S acidic ribosomal protein P0 | RPLP0 | P05388 |
| 40S ribosomal protein S5 | RPS5 | P46782 |
| Ribosome-binding protein 1 | RRBP1 | Q9P2E9 |
| Reticulon-3 | RTN3 | O95197 |
| Succinate dehydrogenase [ubiquinone] iron-sulfur subunit, mitochondrial | SDHB | P21912 |
| Septin-7 | SEPTIN7 | Q16181 |
| Splicing factor 3A subunit 1 | SF3A1 | Q15459 |
| Splicing factor 3B subunit 6 | SF3B6 | Q9Y3B4 |
| Splicing factor, proline- and glutamine-rich | SFPQ | P23246 |
| Monocarboxylate transporter 1 | SLC16A1 | P53985 |
| Excitatory amino acid transporter 1 | SLC1A3 | P43003 |
| Solute carrier family 2, facilitated glucose transporter member 1 | SLC2A1 | P11166 |
| 4F2 cell-surface antigen heavy chain | SLC3A2 | P08195 |
| SAFB-like transcription modulator | SLTM | Q9NWH9 |
| Sorcin | SRI | P30626 |
| Syntaxin-binding protein 1 | STXBP1 | P61764 |
| SURP and G-patch domain-containing protein 2 | SUGP2 | Q8IX01 |
| Synaptophysin | SYP | P08247 |
| Tight junction protein ZO-1 | TJP1 | Q07157 |
| Transmembrane emp24 domain-containing protein 10 | TMED10 | P49755 |
| Lamina-associated polypeptide 2, isoform alpha | TMPO | P42166 |
| Toll-interacting protein | TOLLIP | Q9H0E2 |
| Mitochondrial import receptor subunit TOM70 | TOMM70 | O94826 |
| Nucleoprotein TPR | TPR | P12270 |
| Heat shock protein 75 kDa, mitochondrial | TRAP1 | Q12931 |
| U2 snRNP-associated SURP motif-containing protein | U2SURP | O15042 |
| Ubiquitin carboxyl-terminal hydrolase isozyme L1 | UCHL1 | P09936 |
| UDP-glucose:glycoprotein glucosyltransferase 1 | UGGT1 | Q9NYU2 |
| Vinculin | VCL | P18206 |
| Vimentin | VIM | P08670 |
| Actin-binding protein WASF2 | WASF2 | Q9Y6W5 |
| WD repeat-containing protein 37 | WDR37 | Q9Y2I8 |
| Tyrosine--tRNA ligase, cytoplasmic | YARS1 | P54577 |
| 14-3-3 protein beta/alpha | YWHAB | P31946 |
| BUB3-interacting and GLEBS motif-containing protein ZNF207 | ZNF207 | O43670 |


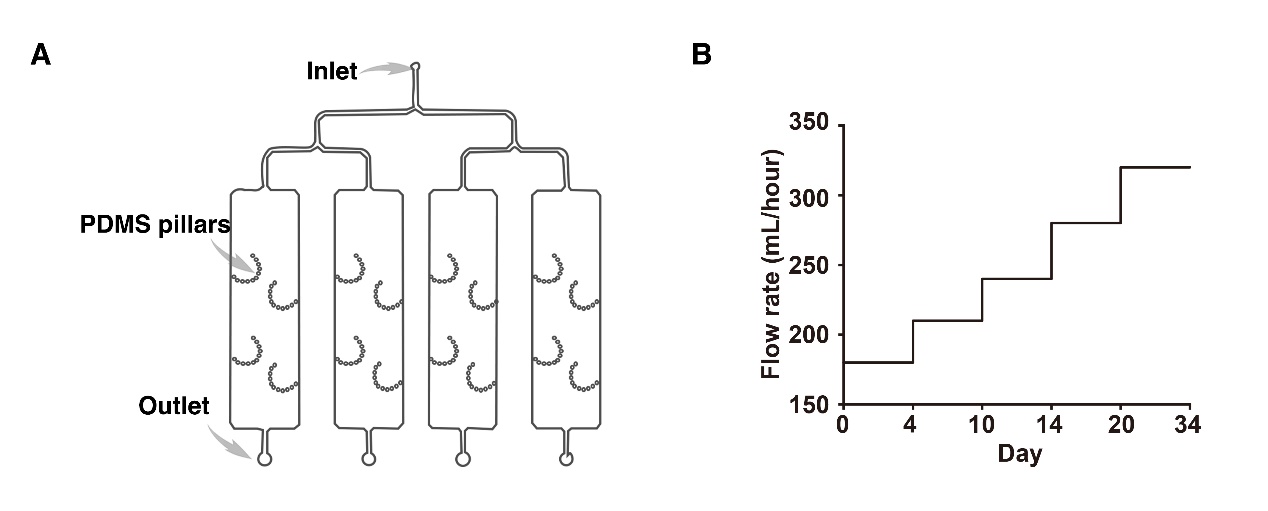


**Fig. S1. Chip design and culture medium injection flow rate. (A)** The detailed design of the microfluidic chip. **(B)** The injection flow rate of the culture media in the inlet on different time points. The initial flow rate starts from 180 µL/hour.


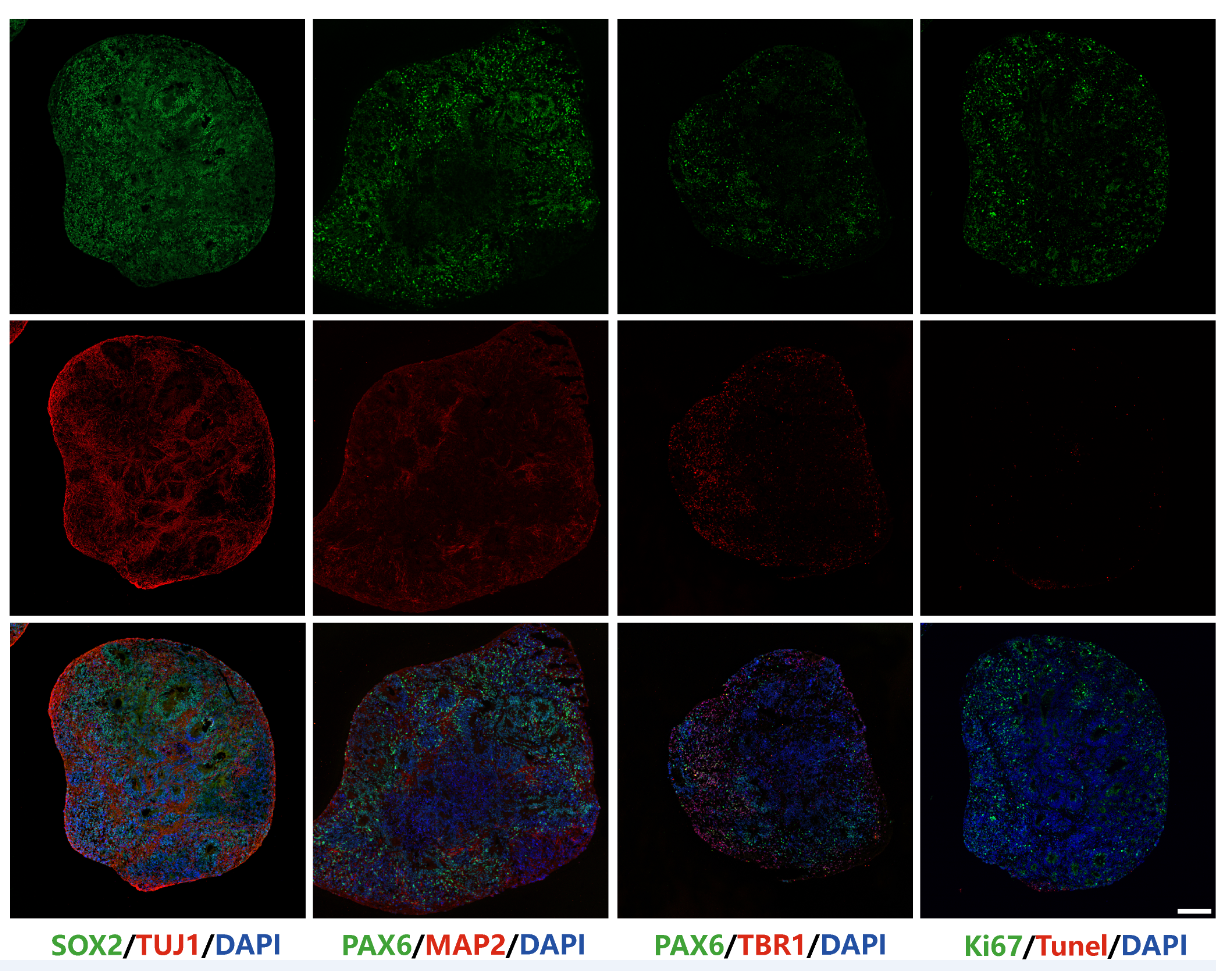


**Fig. S2.** Immunofluorescence staining images of the COs cultured under the 3D multichannel microfluidic platform at day 34. Scale bar is 200 µm.


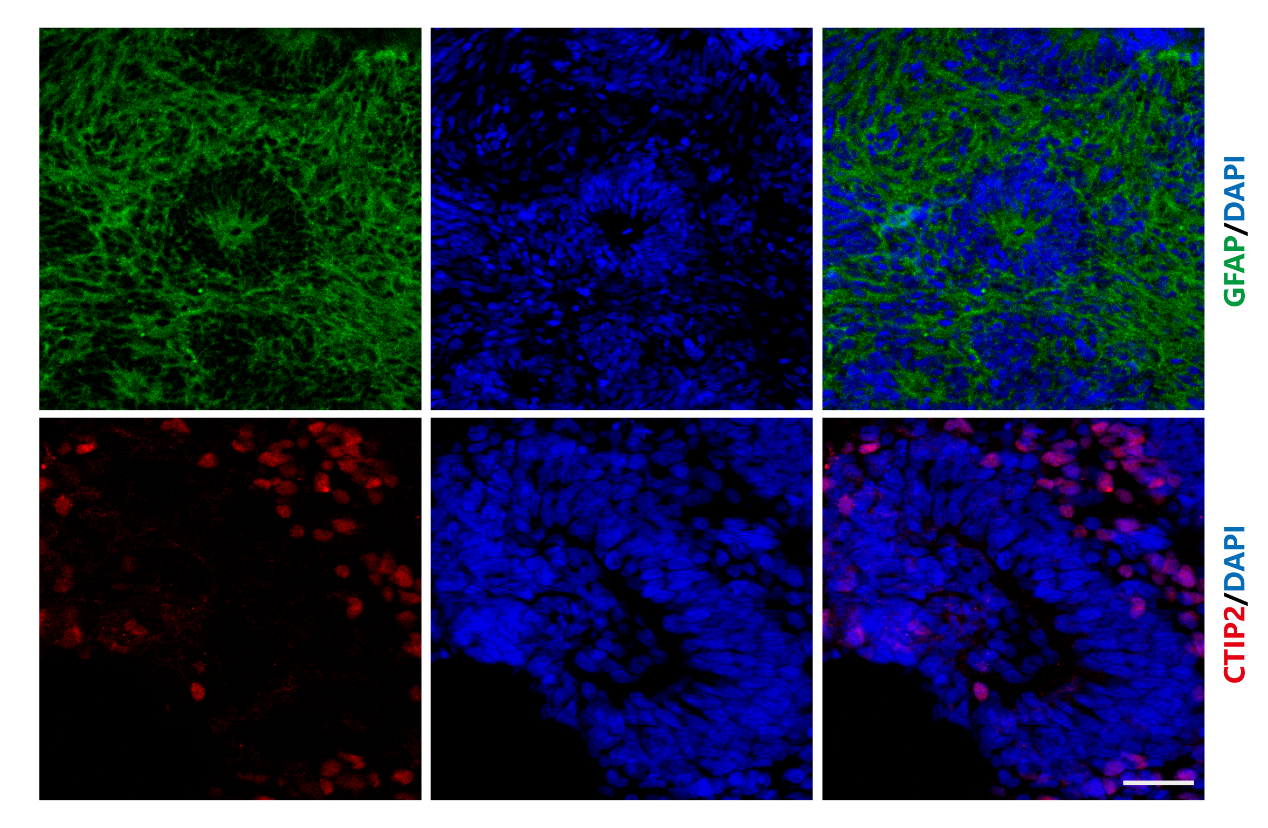


**Fig. S3.** Immunofluorescence staining images of the COs cultured under the 3D multichannel microfluidic platform at day 60. Scale bar is 200 µm.


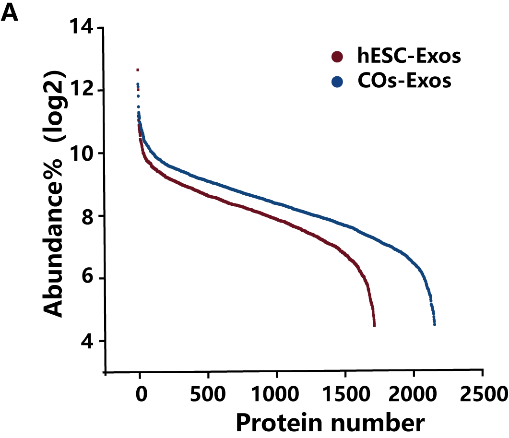


**Fig. S4.** Expression abundance curve of hESC-Exos and COs-Exos.

**
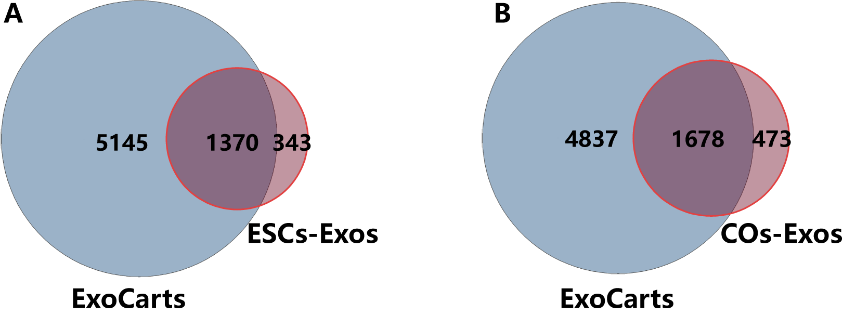
**

**Fig. S5.** **Venn diagram of Exos versus ExoCarta. (A)** Venn diagram of hESCs-Exos versus ExoCarts. **(B)** Venn diagram of COs-Exos versus ExoCarts.


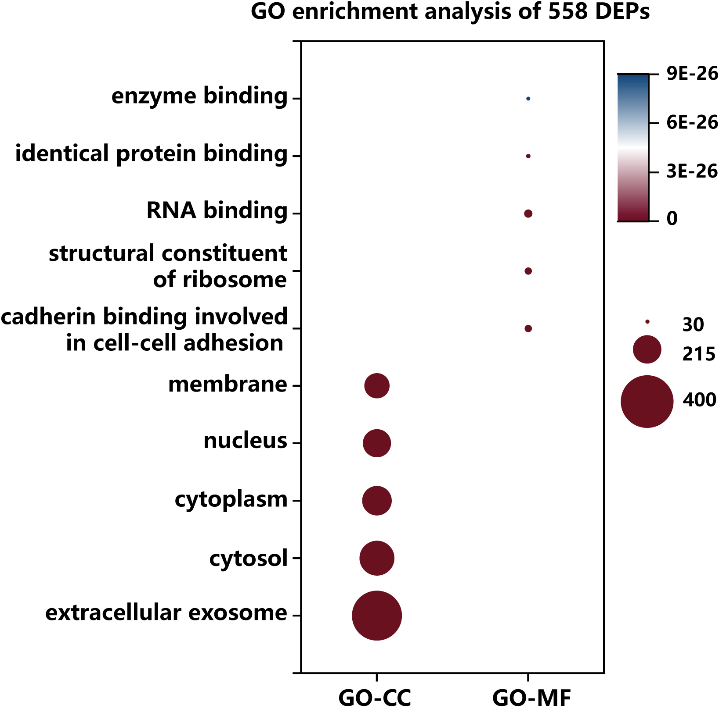


**Fig. S6.** GO enrichment analysis of 558 DEPs commonly expressed in COs-Exos at the four different periods.


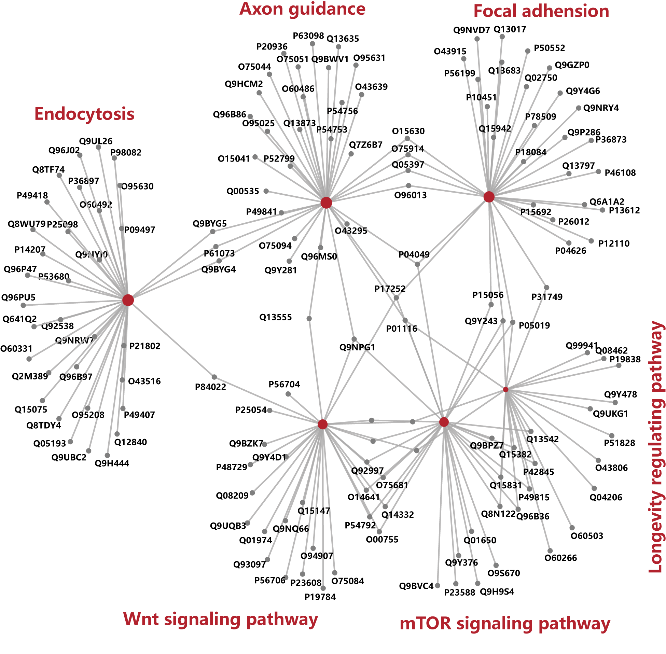


**Fig. S7.** KEGG enrichment analysis of LM-COs-Exos.


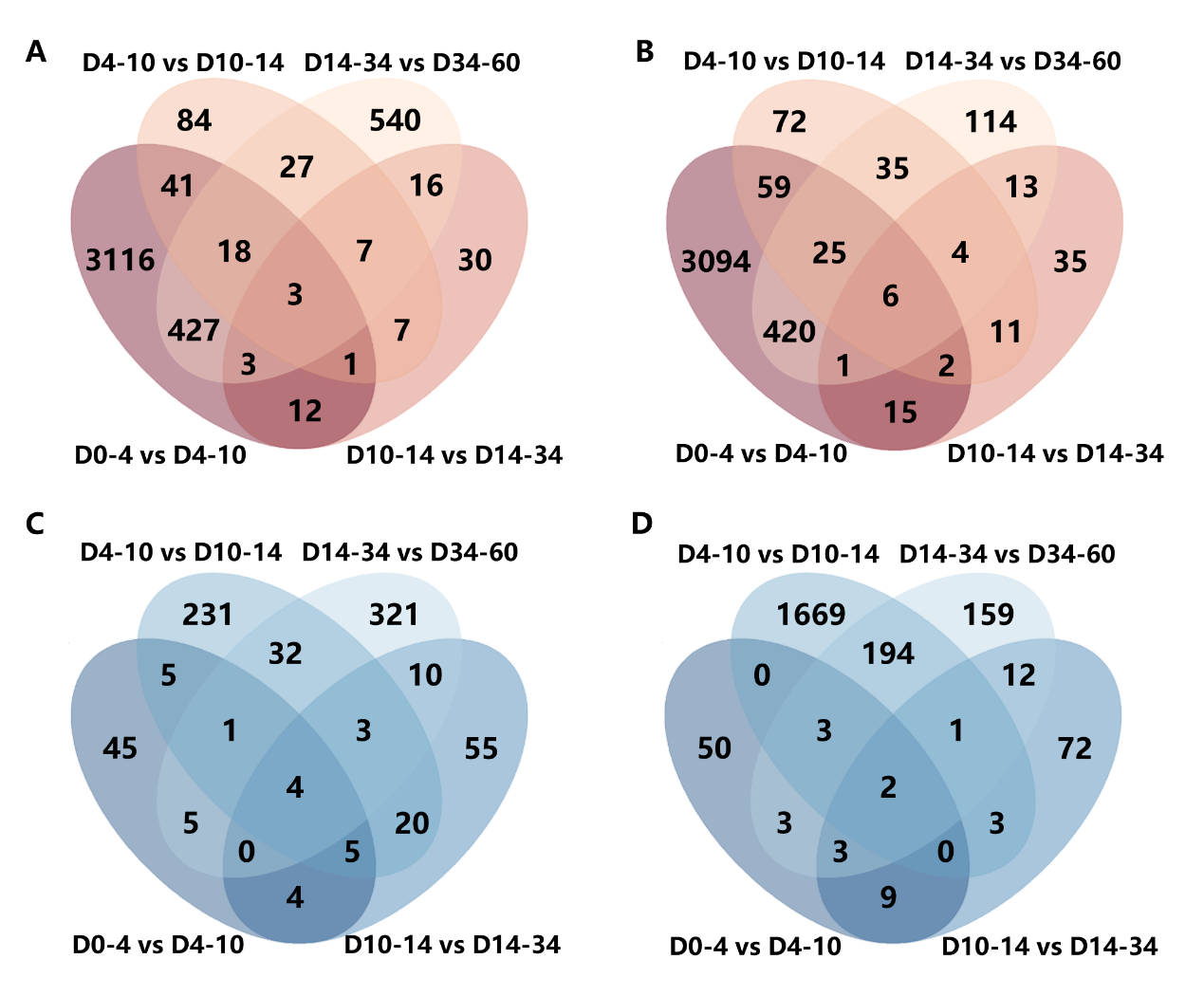


**Fig. S8. (A)** Venn diagram of upregulated proteins in COs-Exos, including proteins with an expression level of 0 on D0-4. **(B)** Venn diagram of upregulated proteins in COs-Exos, including proteins with an expression level of 0 on D0-4 and D4-10. **(C)** Venn diagram of downregulated proteins in COs-Exos, including proteins with an expression level of 0 on D0-4. **(D)** Venn diagram of downregulated proteins in COs-Exos, including proteins with an expression level of 0 on D0-4 and D4-10.
